# Risk assessment and prediction for lung cancer among Hong Kong Chinese men

**DOI:** 10.1186/s12885-022-09678-y

**Published:** 2022-05-28

**Authors:** Lap Ah Tse, Feng Wang, Martin Chi-sang Wong, Joseph Siu-kei Au, Ignatius Tak-sun Yu

**Affiliations:** 1 JC School of Public Health and Primary Care, The Chinese University of Hong Kong, Sha Tin, Hong Kong SAR China; 2 Department of Clinical Oncology, Hong Kong Adventist Hospital, Tsuen Wan, Hong Kong SAR China

**Keywords:** Risk prediction, Lung cancer, Residential radon exposure, Indoor air pollution

## Abstract

**Objective:**

Most of the previous risk prediction models for lung cancer were developed from smokers, with discriminatory power ranging from 0.57 to 0.72. We constructed an individual risk prediction model for lung cancer among the male general population of Hong Kong.

**Methods:**

Epidemiological data of 1,069 histology confirmed male lung cancer cases and 1,208 community controls were included in this analysis. Residential radon exposure was retrospectively reconstructed based on individual lifetime residential information. Multivariable logistic regression with repeated cross-validation method was used to select optimal risk predictors for each prediction model for different smoking strata. Individual absolute risk for lung cancer was estimated by Gail model. Receiver-operator characteristic curves, area under the curve (AUC) and confusion matrix were evaluated to demonstrate the model performance and ability to differentiate cases from non-cases.

**Results:**

Smoking and smoking cessation, education, lung disease history, family history of cancer, residential radon exposure, dietary habits, carcinogens exposure, mask use and dust control in workplace were selected as the risk predictors for lung cancer. The AUC of estimated absolute risk for all lung cancers was 0.735 (95% CI: 0.714–0.756). Using 2.83% as the cutoff point of absolute risk, the predictive accuracy, positive predictive value and negative predictive value were 0.715, 0.818 and 0.674, respectively.

**Conclusion:**

We developed a risk prediction model with moderate discrimination for lung cancer among Hong Kong males. External validation in other populations is warranted for this model in future studies.

**Supplementary Information:**

The online version contains supplementary material available at 10.1186/s12885-022-09678-y.

## Introduction

Lung cancer is the top leading cause of cancer death worldwide. Over the past decade, lung cancer has consistently accounted for approximate one third of all cancer deaths among Hong Kong males [1]. Advanced technology in detection and treatment of lung cancer have not remarkably improved the 5-year survival rate, as more than 50% of lung cancers were diagnosed at an advanced stage and hence the optimal timing for surgical removal was missed [1]. A substantially higher survival rate was demonstrated in patients with an early stage of lung cancer than those with an advanced stage [2]. Low-dose computed tomography (LDCT) has been proven as an effective screening or surveillance strategy for high-risk individuals to reduce their mortalities via detection and early treatment of lung cancer at early stage [3, 4]; however, its cost–benefit and applicability to the general population remains uncertain. Most of the international lung cancer screening guidelines recommend using an ethnicity-specific risk prediction model to cost-effectively identify high-risk population for receiving further medical screening and undergoing timely treatment [5].

Many lung cancer risk prediction models were developed according to Gail model’s concept for different ethnicities and populations, such as the Bach model, the Spitz model and the Liverpool Lung Project (LLP) model [6], and smoking is the most important risk factor that has been involved in all risk prediction models [6]. Family history of lung cancer and history of lung diseases were selected in most of the risk prediction models [6]. However, with the fall of smoking prevalence in the general population, as is the case in Hong Kong, the risk factors with low to moderate carcinogenic potency (such as residential radon exposure and outdoor air pollution) that were previously masked by the dominating effect of smoking might become apparent [7–9]. However, they were not considered in the well-known risk models developed decades ago. In this study, we examined the various risk factors of lung cancer and quantified their contributions to the overall risk of male lung cancer. We then constructed specific risk prediction models based on the various risk factors and finally estimated individualized cancer risk.

## Methods

### Study population and epidemiologic data

The data was derived from an established case–control study for male lung cancer in Hong Kong. Details of the study design was described previously [7]. Briefly, cases were newly diagnosed lung cancer within 3 months and recruited from the largest oncology center of Hong Kong from 2004 to 2006. All cases were histologically confirmed primary carcinoma of the lung (ICD-9-CM code 162). They were aged 35–79 years-old and each case was frequency matched in 5-year age groups by a community control. Any cases or controls with a history of physician-diagnosed cancer in any other sites were excluded. A total of 1208 lung cancer cases and 1069 community controls were included in the final data analysis. This study was approved by the ethics committees of both the Chinese University of Hong Kong and the Queen Elizabeth Hospital of the Hong Kong Hospital Authority (KC/KE 04–0014/ER-1).

Information on socio-demographics, previous history of lung diseases, family history of cancer, lifetime habits of tobacco smoking, indoor air pollution [i.e., environmental tobacco smoke (ETS), incense burning, mosquito coil burning, and cooking fumes] [9], lifetime occupational exposures to known or suspected lung carcinogens [8], lifetime residential history [9], and dietary habits were obtained from face-to-face interview (for lung cancer cases) or telephone interview (for community controls).

### Assessment of residential radon exposure

Cumulative residential radon exposure was assessed using a semi-quantitative score which was calculated according to each participant’s lifetime residential histories (e.g., building materials and wall surface covering materials,, building age, window opening practices, floor level) using information available from a territory-wide indoor radon survey in Hong Kong [9]. A higher score indicated a higher level of exposure to residential radon. Information on the daily frequency, years of burning incense, and mosquito coil at home were also collected.

### Statistical analysis

Before building risk prediction model, we categorized continuous variables as the following: age on interview (< 50 years, 50–59 years, 60–69 years and ≥ 70 years), pack-years of smoking (< 20, 20–39 and ≥ 40 pack-years), years of smoking cessation (2–5 years, 5–10 years, 10–20 years and ≥ 20 years), score of residential radon exposure (< 8.11, 8.11 to < 8.64, 8.64 to < 9.77, and ≥ 9.77, based on the quartile distribution of scores among control) and alcohol drinking (non-drinkers, < 4 times/week and ≥ 4 times/week). All analyses were performed by using R software Version 4.0.2 (The R Foundation).

#### Risk prediction model building

**Fig. 1:**
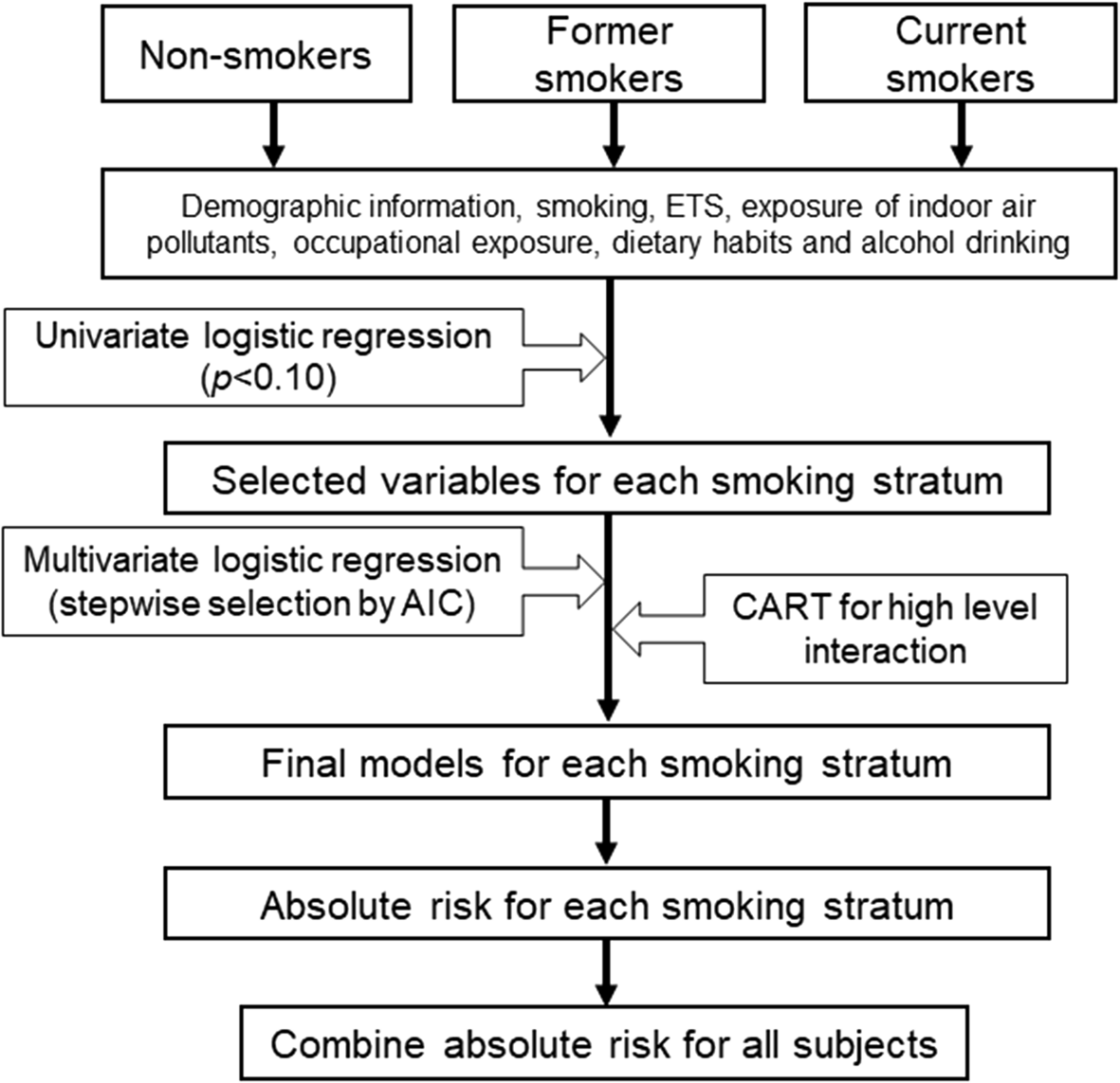
Flow chart for building risk prediction model

We adopted Sptiz’s method to build risk prediction model [10] and estimate absolute risk of lung cancer based on the method of Gail et al. [11]. Detailed method was described in Fig. 1 and supplementary materials S1. Two steps were used to refine the risk predictors by smoking status. Firstly, potential risk predictors were selected by using univariate logistic regression model. Then a tenfold, 10 times repeated cross-validation method of multivariable logistic regression with stepwise selection procedure was used to build three risk models for the three smoking strata.

#### Absolute risk calculation and performance evaluation

The third step was to estimate the absolute risk of lung cancer by using the odds ratios derived from risk models, age-specific incidence rate of male lung cancer, and mortality rate from causes other than lung cancer (Appendix Table 1). Absolute risk unified the estimated risks from three risk models for different smoking status and made them comparable by using the formula *v*_*i*_ = *c*_i_*I, where *c*_*i*_ was an adjustment constant for each smoking status group (Appendix Table 2), and *I* was the age-specific incidence. R package “iCARE” (version 1.16.0) was used to calculate absolute risk [12].

**Table 1:** Distribution of study population by age and smoking status

		Control (*N* = 1069)	Cases (*N* = 1208)	*p* value^*^
Age (year-old)	Mean ± SD^#^	66.2 ± 0.3	65.8 ± 0.3	0.326
	< 50	92 ( 8.6)	98 ( 8.1)	0.060
	50–60	167 (15.6)	206 (17.1)	
	60–70	326 (30.5)	419 (34.7)	
	≥ 70	484 (45.3)	485 (40.1)	
Education	Primary school or below	142 (13.5)	298 (25.1)	< 0.001
	Middle school	383 (36.4)	525 (44.2)	
	College or above	528 (50.1)	366 (30.8)	
Marital status	Married	183 (17.3)	198 (16.6)	0.646
	Others	876 (82.7)	998 (83.4)	
Monthly income (HKD)	< 4000	456 (42.7)	735 (60.8)	< 0.001
	> 4000	285 (26.7)	306 (25.3)	
	No answer	328 (30.7)	167 (13.8)	
History of lung diseases	Yes	112 (10.5)	286 (23.7)	< 0.001
Cancer history in first-degree relatives	Yes	134 (12.5)	239 (19.8)	< 0.001
Smoking				
Smoking status n (%)	Never	536 (50.1)	132 (10.9)	< 0.001
	Former	357 (33.4)	340 (28.1)	
	Current	176 (16.5)	736 (60.9)	
Pack-years	Non-smokers	547 (51.2)	140 (11.6)	< 0.001
	< 20	172 (16.1)	122 (10.1)	
	20–40	171 (16.0)	317 (26.2)	
	≥ 40	179 (16.7)	629 (52.1)	
Smoking cessation	Smoker	176 (16.7)	736 (61.4)	< 0.001
	2–5 years	45 ( 4.3)	81 ( 6.8)	
	5–10 years	55 ( 5.2)	65 ( 5.4)	
	10–20 years	100 ( 9.5)	98 ( 8.2)	
	≥ 20 years	145 (13.7)	87 ( 7.3)	
	non-smokers	536 (50.7)	132 (11.0)	
ETS exposure	No	281 (26.3)	230 (19.1)	< 0.001
	Yes	788 (73.7)	977 (80.9)	
Exposure of indoor air pollutants				
Radon	Mean ± SD	8.79 ± 1.30	8.93 ± 1.22	0.008
	Median (range)	8.86 (5.46–13.00)	8.86 (5.17–14.00)	
	< 25 (5.17–8.11)	263 (24.6)	230 (19.0)	0.005
	25–50 (8.11–8.64)	261 (24.4)	301 (24.9)	
	50–75 (8.64–9.77)	277 (25.9)	318 (26.3)	
	> 75 (9.77–13.00)	268 (25.1)	359 (29.7)	
Incense burning at home	Yes	590 (55.2)	776 (64.2)	< 0.001
Mosquito coil burning	Yes	160 (15.1)	220 (18.3)	0.036
PM10 exposure				
Annual average exposure	Mean ± SD	73.1 ± 0.4	75.7 ± 0.3	< 0.001
	Median (range)	73.22 (35.89–117.27)	75.45 (44.43–110.30)	
Occupational exposure				
Carcinogen exposure	Yes	453 (42.4)	624 (51.7)	< 0.001
Mask used in workplace	Yes	112 (10.5)	116 ( 9.6)	0.488
Ventilation in workplace	Yes	976 (91.3)	1065 (88.2)	0.014
Dust control in workplace	Yes	1022 (95.6)	1009 (83.5)	< 0.001
Dietary habits and drinking habits				
Fruit/green tea	≥ 1 time /day	422 (39.5)	442 (36.6)	0.157
Meat	≥ 1 time /day	253 (23.7)	128 (10.6)	< 0.001
Preserved food	≥ 1 time /day	198 (18.5)	325 (26.9)	< 0.001
Alcohol drinking	Non-drinkers	551 (51.5)	451 (37.4)	< 0.001
	< 4 times /week	314 (29.4)	331 (27.4)	
	≥ 4 times /week	204 (19.1)	425 (35.2)	

^*^ *p* value from the two-sided chi-square test for categorical variables and Student’s t test for continuous variables

^#^ *SD*, Standard deviation

**Table 2:** Multivariable logistic regression analysis for lung cancer by smoking status^a^

		Odds ratio (95% CI)		
		Never smokers (132/536) ^b^	Former smokers (340/357) ^b^	Current smokers (736/176) ^b^
Education	Primary school or below			
	Middle school		0.50 (0.33–0.76)	
	College or above		0.37 (0.23–0.59)	0.49 (0.35–0.70)
Marital status	Married			
	Others		0.57 (0.36–0.90)	
History of lung diseases		2.82 (1.53–5.12)	2.28 (1.53–3.45)	
Cancer history in first-degree relatives		2.44 (1.50–3.94)	1.56 (0.99–2.48)	2.25 (1.32–4.09)
Smoking status				
Pack-years	< 20	N.A		
	20–40		1.90 (1.24–2.91)	2.40 (1.36–4.22)
	≥ 40		2.41 (1.55–3.74)	3.09 (1.80–5.26)
Years of smoking cessation	2–5 years	N.A		N.A
	5–10 years		0.60 (0.35–1.02)	
	10–20 years		0.65 (0.40–1.03)	
	≥ 20 years		0.51 (0.31–0.83)	
Exposure of indoor air pollutants				
Residential radon exposure (in quartiles)^c^	First			
	Second	2.42 (1.25–4.85)		
	Third	2.31 (1.21–4.57)		
	Fourth	2.91 (1.55–5.68)		
Occupational exposure				
Carcinogen exposure		1.84 (1.22–2.77)		1.33 (0.93–1.89)
Mask used in workplace			0.50 (0.28–0.88)	
Dust control in workplace		0.31 (0.16–0.67)	0.22 (0.12–0.40)	0.20 (0.08–0.41)
Dietary habits and drinking habits				
Fruit/green vegetable	(≥ 1 time /day)	1.44 (0.95–2.19)		
Meat	(≥ 1 time /day)	0.47 (0.26–0.80)	0.61 (0.40–0.91)	0.46 (0.26–0.83)

^a^ Variables was selected by stepwise selection procedure by minimal *AIC*; *CI*, Confidence interval

^b^ no. of cases / no. of controls

^c^ Using the quartile score of community referents as the cut point (first, second, third, and fourth quartile: (< 8.11, 8.11 to < 8.64, 8.64 to < 9.77, and ≥ 9.77, respectively) to classify different levels of radon exposure

CART analysis (“rpart” method) was used to identify the cut-off points on absolute risk to group the participants into low, medium and high risk or low/high risk groups. Then confusion matrix analysis from “Caret” package was used to evaluate the discriminative power of estimated absolute risk.

#### Comparison with Spitz’s model

We re-calibrated Spitz model using the current data and variables included in the Spitz model to compare the discriminative power between our Hong Kong male lung cancer (HKMLC) model and the Spitz model. The risk predictors retained in the Spitz model were ETS and family history for never smokers; Emphysema, dust exposure, family history, age stopped smoking, and hay fever for former smokers; Emphysema, dust exposure, asbestos exposure, family history, pack-years, and hay fever for current smokers. Hay fever was excluded from the recalibration because it was not available from current data. The dust and asbestos exposure were involved in one risk predictor—carcinogen exposure. Therefore, we used carcinogen exposure instead of these two factors in the model.

All risk factors listed above were forced into a logistic regression model with 10 times repeated tenfold cross-validation, and calculated their odd ratios. Absolute risk derived from re-calibrated Spitz model was calculated using the same R package “iCARE”. Discrimination ability between the HKMLC model and Spitz model was compared by calculating the area under the curve (AUC) based on the receiver operating characteristic analysis.

#### Sensitivity analysis by histological subtypes and radon

To explore the potential difference in the risk predictors among histological subtypes of lung cancer, odds ratios of candidate risk predictors were calculated by using univariate logistic regression. Residential radon exposure was forced adding/removing to each final model stratified by smoking status to investigate its contribution to model performance.

## Results

A total of 1208 male patients with lung cancer and 1069 control subjects were included for this analysis. As shown in Table 1, the mean age of cases and controls was comparable. Lung cancer cases had significantly higher prevalence of current smoking (60.9% *vs.* 16.5%) but lower prevalence of smoking cessation (28.1% vs. 33.4%) than that of the controls. The distribution of education levels, monthly income, history of lung diseases, cancer history in first-degree relatives, ETS exposure, residential radon exposure, incense burning at home, PM10 exposure, carcinogen exposure in workplace, meat intake, preserved food intake, and alcohol drinking are significantly different between lung cancer cases and controls. All variables listed in Table 1 were included in the selection of predictors for risk models.

### Selection of predictors for risk models

We performed univariate analyses for each subgroup stratified by smoking status and included the potential risk predictors with *p* < 0.10 in the multivariable model (Appendix Table 3). Table 2 summarises the predictors retained in the multivariable logistic regression model, which included history of lung disease, cancer history in first-degree relatives, residential radon exposure, carcinogen exposure in workplace, dust control in workplace, having fruit/green vegetable ≥ 1 time /day, and having meat ≥ 1 time /day for never smokers. Among former smokers, educational level, marital status, history of lung disease, cancer history in first-degree relatives, smoking pack-years, years of smoking cessation, mask use and dust control in workplace, and having meat ≥ 1 time /day were kept in the final model. Among current smokers, education, cancer history in first-degree relatives, pack-years of tobacco smoking, carcinogen exposure and dust control in workplace, and having meat ≥ 1 time /day were kept in the final model. The same predictors kept in each final model were also identified in the decision trees of the CART models. No higher order interactions were evident from these models (data not shown).

**Table 3:** Model performance by all data

Smoking category	*p* from Hosmer-Lemeshow goodness of fit	AUC^†^ (95% CI)	Concordance statistic (95% CI)^‡^
Never smokers	0.493	0.583 (0.550–0.617)	0.71 (0.67–0.77)
Former smokers	0.260	0.681 (0.646–0.715)	0.74 (0.72–0.79)
Current smokers	0.502	0.532 (0.512–0.553)	0.71 (0.67–0.76)

^†^ *AUC*, Area under curve by the receiver operating characteristic curve

^‡^ Derived from 999-fold cross-validation of combined dataset

### Model performance

As illustrated in Table 3, the risk models were well calibrated throughout the entire range of probabilities, as indicated by the non-statistically significant Hosmer–Lemeshow goodness-of-fit test statistics (0.493 for never smokers, 0.260 for former smokers, and 0.502 for current smokers). The AUC statistic obtained from the combined set was low for never smokers (AUC 0.583, 95% CI 0.550–0.617) and current smokers (AUC 0.532, 95% CI 0.512–0.553) but it was higher for former smokers (AUC 0.681, 95% CI 0.646–0.715). The C statistics calculated by 999-fold cross-validation of the combined dataset for never, former and current smokers were 0.71 (95% CI 0.67–0.77), 0.74 (95% CI 0.72–0.79) and 0.71 (95% CI 0.67–0.76), indicating that the risk models performed reasonably well in discriminating between case patients and control subjects.

### Risk models with/without residential radon exposure

We tested the contribution of residential radon exposure in all three risk models as it was included in the model of never smokers. After adding/removing residential radon exposure to each final model stratified by smoking status, the AUC was increased in never smokers, decreased in former smokers and no change in current smokers (Appendix Table 4).

**Table 4:** Model performance of estimated absolute risk of lung cancer (by 2.83%)^a^

Smoking category	Accuracy	Positive predictive value	Negative predictive value
Never smokers	0.826 (0.795–0.854)	0.674	0.838
Former smokers	0.489 (0.452–0.527)	0.489	1.000
Current smokers	0.807 (0.780–0.832)	0.821	0.500
Overall	0.715 (0.696–0.734)	0.818	0.674

^a^ Results from confusion matrix

### Estimation of absolute risk for male lung cancer

**Fig. 2:**
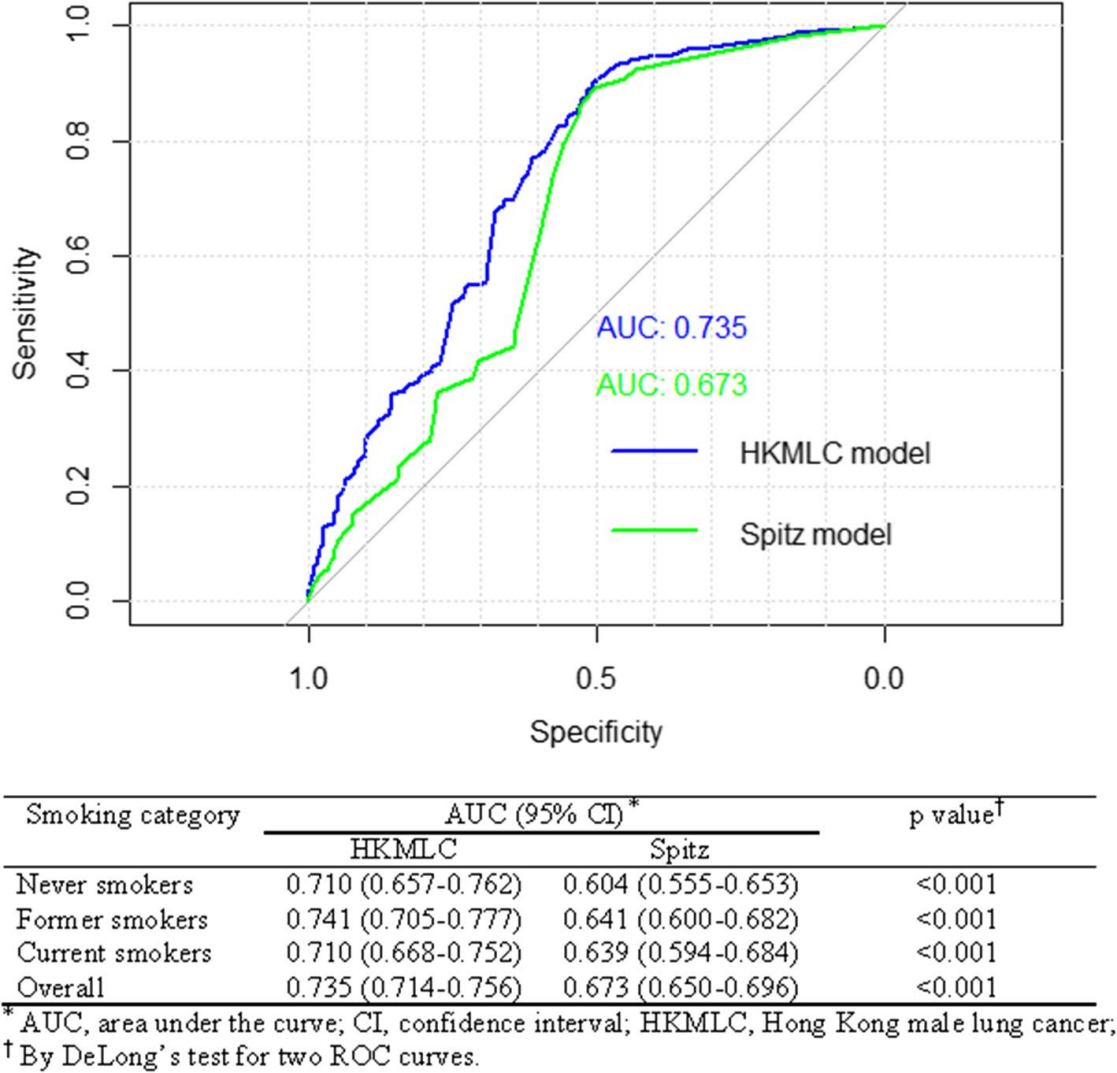
AUC comparison of estimated absolute risk between HKMLC model and Spitz model

Absolute risk of lung cancer was estimated by Spitz’s method, which allowed us to combine results from three risk prediction models together. The model of absolute risk showed a moderate discriminative power with AUC of 0.735 (95% CI, 0.714–0.756) (Fig. 2). Comparing with the re-calibrated Spitz model, the discrimination ability of HKMLC model was improved significantly, which was applicable to each smoking stratum and overall subjects (*p* < 0.01) (Fig. 2).

**Fig. 3:**
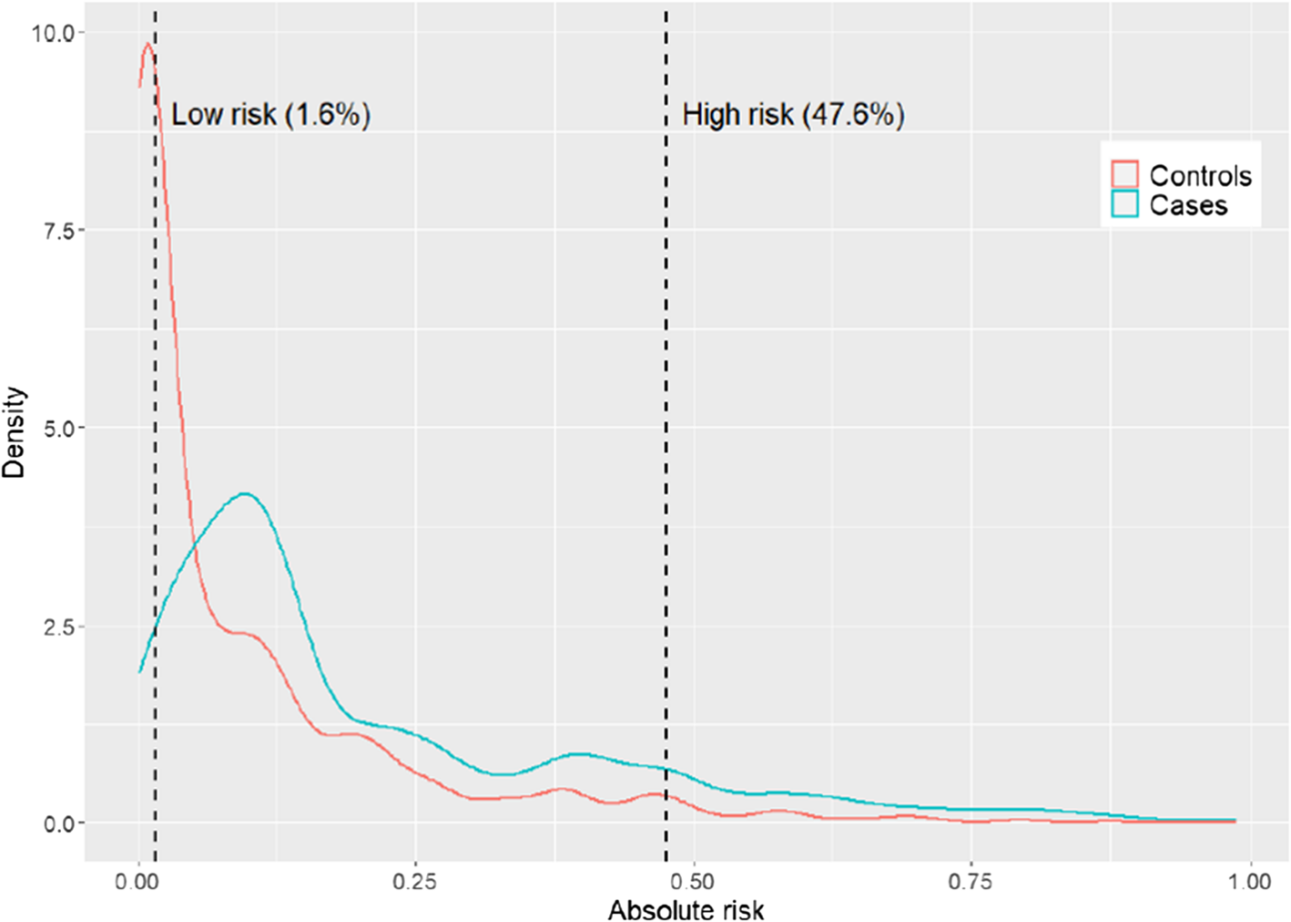
Distribution of estimated absolute risk of lung cancer among cases and controls

The distribution of estimated absolute risk was shown in Fig. 3. By CART analysis (“rpart” method), all subjects were grouped to the low, medium and high risk of lung cancer according to the cut-off points of 1.6% and 47.6%. When compared with the low risk group, the model showed a good discriminative power to identify males with high risk of lung cancer with the accuracy, sensitivity and specificity of 0.850, 0.643 and 0.944, respectively.

The median value of estimated absolute risk among controls of this study was 2.83%. Based on the cut-off point 2.83%, the accuracy, positive predictive value and negative predictive value were 0.715 (95% CI, 0.696–0.734), 0.818 and 0.674, respectively (Table 4). The accuracy was more than 0.80 for never smokers and current smokers. The predictive accuracy of former smokers was lower than 0.500, because the estimated absolute risk was higher than 2.83% for most of the former smokers (696 out of 697 subjects).

### Risk predictors for histological sub-types of lung cancer

Appendix Table 5-7 showed the risk predictors for different histological subtypes of lung cancer. Almost all cases of squamous cell carcinoma or small cell lung cancer were current or former smokers. As cigarette smoking showed a dominant contribution to the risk of these subtypes, relatively fewer other risk predictors were retained in the final model including education, history of lung disease, cancer history, mask use and dust control in workplace. The pattern of risk predictors for adenocarcinoma was similar to that for all lung cancer cases in different smoking subgroups.

## Discussion

We developed a risk prediction model of lung cancer for Hong Kong males with moderate discrimination ability. The model has the potential to identify individuals with high risk by epidemiological and clinical information. This risk model appears to discriminate between high and low risk satisfactorily, although it needs an external validation.

Nine epidemiological models for risk prediction of lung cancer were published since 2003. Their discriminative powers (AUC) were from 0.530 to 0.859. Liverpool Lung Project (LLP) model and Spitz model are the only two models that could be applied to the never smokers [6]. Only Spitz model solo showed the discrimination power of absolute risk for never smokers with AUCs of 0.57 (95%CI 0.47–0.66) [10] which was much lower than the AUCs of 0.710 (95%CI 0.657–0.762) from HKMLC model. Whilst other models with higher discriminative power were developed among ever smokers. Smoking intensity, family history of cancer, non-malignant lung disease history, and occupational exposure to carcinogens especially asbestos are the common risk predictors for HKMLC, LLP and Spitz models [6]. In addition, HKMLC model included more risk predictors such as education, dietary habits, and residential radon exposure, which explained a significantly improved discrimination of HKMLC model for estimated absolute risk. Previously, researchers tried to use the simplest models for cancer risk prediction because the clinicians have less time to perform the data collection and risk calculation [10, 13]. However, with the improvement of online survey and human–computer interface, interview and risk calculation is no longer a barrier for the utility of sophisticated risk prediction model such as the HKMLC model.

A strength of HKMLC model is the improvement on discriminative power for never smokers. The AUC was improved from 0.604 to 0.710 comparing with Spitz model. As we know, smoking is a predominant risk factor for lung cancer and most cases of lung cancer are smokers. Most risk prediction models were developed for ever smokers and of little doubt, smoking played a key role in these risk models. However, smoking rate has dropped continuously in recent decades, which prompted the researchers to shift their attentions to the etiology of lung cancer among never smokers. The focus is more on some environmental risk factors with low or moderate potency of carcinogenicity, such as environmental tobacco smoke, indoor and outdoor air pollution, and dietary habits [14]. Residential radon exposure causes approximately 21,000 deaths annually from lung cancer, making it the second most important cause of lung cancer after smoking, but it is usually neglected by researchers [15]. To the best of our knowledge, this is the first study to consider residential radon exposure as a predictor for lung cancer risk. The present study also explored the possible contribution of ambient PM10 exposure in the risk model.

Prediction models have recently changed focus to include genetic markers and/or clinical assessment, as well as attempts to further improve overall performance. Several to tens of single nucleotide polymorphisms were added to prediction models such as the extend LLP model, Gene-Based Risk Score, Chinese Multifactorial Genetic Model, and Gene Variants in African Americans Model et al. [6]. However, there were no universal improvements in discrimination compared with models built on epidemiological data alone. Epidemiological plus clinical assessment models were also explored for lung cancer risk prediction such as the Extended Bach Model, Korean Men Model, Pulmonary Function With Lung Response Model, Two-Stage Clonal Expansion Models and Extended Spitz Model. Several clinical assessment models were also studied, including LDCT, pulmonary function, DNA capacity and clinical traits. However, the improvement of model performance was very limited [6].

There were several limitations of this study. Firstly, the number of cases and controls were quite uneven after stratification by smoking as the matching criterion of this case–control study was age only. The nature of more smokers among lung cancer cases caused the small number of lung cancer cases in never smokers and small number of controls in current smokers. The small samples size in some strata might lead to unstable results although we used repeated cross-validation methods. Also, some subgroups of potential risk factor were small and might be excluded by predictors selection process. Secondly, the model was developed among males and it may not be directly applied to the females without a further validation. Thirdly, the HKMLC model might only have internal validity. External validation especially in large prospective cohorts in other geographical areas is warranted to examine the reliability and generalizability of the findings. Fourthly, our dataset didn’t provide the complete data of dietary quality and quantity. The frequency of meat, fruit/green vegetable was an alternative, which limit the evaluation on the role of dietary in current model.

In conclusion, the current study developed a lung cancer prediction model with moderate discrimination and residential radon exposure improved the discrimination power for never smokers. Although this newly developed model demonstrated a relatively higher discriminative accuracy than those developed in many other populations, we recommend external validation of this model in other populations.

## Supplementary Information

**Additional file 1.**

## Data Availability

The datasets generated and/or analysed during the current study couldn’t be shared publicly because this condition isn’t included in the written consent. Researchers could contact the corresponding author if they are interested in this dataset.

## Abbreviations

LDCT: Low-dose computed tomography
LLP: Liverpool Lung Project
ETS: Environmental tobacco smoke
HKMLC: Hong Kong male lung cancer
AUC: Area under the curve
