# Supplementary material for: Risk assessment and prediction for lung cancer among Hong Kong Chinese men
Source: BMC Cancer. 2022 May 28;22:585. doi: 10.1186/s12885-022-09678-y (PMC9145456; doi:10.1186/s12885-022-09678-y)
Supplement: Supplementary file 1 — Additional file 1. [file 12885_2022_9678_MOESM1_ESM.docx]

**Supplementary materials S1** Method for risk prediction model building and absolute risk estimation

We adopted Sptiz’s method to build risk prediction model using three steps involving refining the risk predictors and evaluating the model performance, as described in Figure 1 [1]. Firstly, we divided all participants into three strata by smoking status (never smokers, former smokers and current smokers). We then performed univariate logistic regression analysis in each smoking stratum with these variables: age, educational level, marital status, lung disease history, family history of cancer, smoking, Environmental Tobacco Smoke (ETS) exposure, residential radon exposure, occupational hazards exposure and protection equipment usage, dietary habit and alcohol drinking. Variables with *p*<0.10 were considered as potential risk predictors for further model building.

Secondly, a multivariable logistic regression model with stepwise selection procedure was used to build the final risk models and assess the performance for the three smoking strata. A 10-fold, 10 times repeated cross-validation method (R package “Caret” version 6.0.86) was used to obtain a stable result. CART analysis (“rpart” method) was used to evaluate different order (two-way and above) interactions. Each of the three models was evaluated by the Hosmer - Lemeshow goodness-of-fit test (by R package “ResourceSelection” version 0.3.5). Model discriminative ability was evaluated by the Receiver Operating Characteristic (ROC) curve with the area under the curve (AUC) (by R package “pROC” version 1.16.2). For each risk model, we also calculated the concordance statistic (C-index), which is an index of the model’s discriminative ability similar to the AUC. Bootstrap method where the step was repeated 999 times was used to compute the average C-index and the 95% confidence intervals (95% CI) (by R package “boot” version 1.3.25 and “DescTools” version 0.99.38). Potential difference in the risk models among histological subtypes was compared by repeating the steps for adenocarcinoma, squamous cell carcinoma and small cell lung cancer, respectively.

Thirdly, we estimated the absolute risk of lung cancer by the final risk models based on the method of Gail et al [2]. We obtained the age-specific incidence and mortality of male lung cancer between 2004 and 2006 from the Hong Kong Cancer Registry [3], and treated them as the baseline hazards. The overall mortality rate for the general male population during 2004-2006 was obtained from the Hong Kong Census and Statistics Department [4]. The average age-specific mortality rate from causes other than lung cancer was calculated by subtracting lung cancer mortality from overall male mortality (Appendix table 1). Same as Spitz et al, we also used odds ratios of the individual risk predictors from the final model as relative risks under the rare disease assumption of lung cancer [1]. We then followed the method of Spitz et al to calculate the age and smoking-specific incidence rate of lung cancer based on the formula *v_i_* = *c_i_*I*, where *ci* was an adjustment constant for each smoking status group (Appendix table 2), and *I* was the age-specific incidence. R package “iCARE” (version 1.16.0) was used to calculate the absolute risk [5].

CART analysis (“rpart” method) was used to identify the cut-off points on absolute risk to group the participants into low, medium and high risk or low/high risk groups. Then confusion matrix analysis from “Caret” package was used to evaluate the discriminative power of estimated absolute risk which was shown as accuracy, positive predictive value and negative predictive value.

**References**

[1] M.R. Spitz, W.K. Hong, C.I. Amos, X. Wu, M.B. Schabath, Q. Dong, S. Shete, C.J. Etzel, A risk model for prediction of lung cancer, J Natl Cancer Inst 99(9) (2007) 715-26.

[2] M.H. Gail, L.A. Brinton, D.P. Byar, D.K. Corle, S.B. Green, C. Schairer, J.J. Mulvihill, Projecting individualized probabilities of developing breast cancer for white females who are being examined annually, J Natl Cancer Inst 81(24) (1989) 1879-86.

[3] Hong Kong Cancer Registry, Hong Kong Cancer Regeistry. <http://www3.ha.org.hk/cancereg/default.asp>.

[4] C.a.S.D.o.H. Kong, The Mortality Trend in Hong Kong, 1986 to 2018. <https://www.censtatd.gov.hk/hkstat/sub/sp160.jsp?productCode=FA100094>.

[5] P. Pal Choudhury, P. Maas, A. Wilcox, W. Wheeler, M. Brook, D. Check, M. Garcia-Closas, N. Chatterjee, iCARE: An R package to build, validate and apply absolute risk models, PLoS One 15(2) (2020) e0228198.

Appendix table 1 Lung cancer incidence rates/100 000 and mortality from other causes excluding lung cancer /100 000 (excluding lung cancer mortality) by age

| Age group | Incidence ^1^ | Mortality ^2^ |
| --- | --- | --- |
| 0-4 | 0.00 | 76.65 |
| 5-9 | 0.00 | 9.88 |
| 10-14 | 0.16 | 11.07 |
| 15-19 | 0.00 | 23.50 |
| 20-24 | 0.00 | 41.15 |
| 25-29 | 0.15 | 64.71 |
| 30-34 | 3.45 | 73.57 |
| 35-39 | 8.64 | 97.41 |
| 40-44 | 13.55 | 128.72 |
| 45-49 | 34.01 | 194.63 |
| 50-54 | 69.77 | 322.16 |
| 55-59 | 118.39 | 499.49 |
| 60-64 | 227.71 | 861.20 |
| 65-69 | 318.78 | 1385.98 |
| 70-74 | 471.48 | 2351.76 |
| 75-79 | 652.21 | 4082.01 |
| 80-84 | 734.22 | 6997.66 |
| 85+ | 667.95 | 12958.97 |

1 Hong Kong Cancer Registry, Hong Kong Cancer Regeistry. <http://www3.ha.org.hk/cancereg/default.asp>.

2 C.a.S.D.o.H. Kong, The Mortality Trend in Hong Kong, 1986 to 2018. https://www.censtatd.gov.hk/hkstat/sub/sp160.jsp?productCode=FA100094.

Appendix table 2 Adjustment constants (*c_i_*) to estimate smoking status-specific incidence rates

| Distribution | Lung cancer patients | General population | *c_i_* |
| --- | --- | --- | --- |
| Never smokers | 10.9% | 69.1% | 0.158 |
| Former smokers | 28.2% | 4.8% | 5.875 |
| Current smokers | 60.9% | 26.1% | 2.333 |

Appendix table 3 Univariate analysis of lung cancer risk factors (*p*<0.10) by smoking status^*^

|  | | | | | | Never smokers  (132/536) ^a^ | Former smokers  (340/357) ^a^ | Current smokers  (736/176) ^a^ |
| --- | --- | --- | --- | --- | --- | --- | --- | --- |
| Age groups  (years) | | <50 | | | |  |  |  |
|  |  | 50-60 | | | |  |  |  |
|  |  | 60-70 | | | |  |  |  |
|  |  | ≥70 | | | | 0.021 |  |  |
| Education | Primary school or below | | | | |  |  |  |
|  | Middle school | | | | |  | 0.001 |  |
|  | College or above | | | | |  | <0.001 | <0.001 |
| Marital status | | | | | Married |  |  |  |
|  |  |  |  |  | Others |  | 0.064 |  |
| History of lung diseases | | | | | | <0.001 | <0.001 | 0.013 |
| Cancer history in first-degree relatives | | | | | | <0.001 | 0.024 | 0.003 |
| Smoking and ETS | | | | | | | | |
| Pack-years | | <20 | | | | N.A. |  |  |
|  |  | 20-40 | | | | N.A. | 0.005 | 0.001 |
|  |  | ≥ 40 | | | | N.A. | <0.001 | <0.001 |
| Years of smoking cessation | | | | 2-5 years | | N.A. |  | N.A. |
|  |  |  |  | 5-10 years | | N.A. |  | N.A. |
|  |  |  |  | 10-20 years | | N.A. | 0.031 | N.A. |
|  |  |  |  | ≥ 20 years | | N.A. | <0.001 | N.A. |
| ETS exposure | | | | | |  |  |  |
| Exposure of indoor air pollutants | | | | | | | | |
| Quantiles of radon | | Q1 (5.17-8.11) | | | |  |  |  |
|  |  | Q2 (8.11-8.64) | | | | 0.015 |  |  |
|  |  | Q3 (8.64-9.77) | | | | 0.009 |  |  |
|  |  | Q4 (9.77-13.00) | | | | <0.001 |  |  |
| Incense burning at home | | | | | |  | 0.014 |  |
| Mosquito coil burning | | | | | | 0.097 |  |  |
| Occupational exposure | | | | | | | | |
| Carcinogen exposure | | | | | | <0.001 |  | 0.034 |
| Mask used in workplace | | | | | |  | 0.019 |  |
| Ventilation in workplace | | | | | |  |  |  |
| Dust control in workplace | | | | | | 0.001 | <0.001 | <0.001 |
| Dietary habits and drinking habits | | | | | | | | |
| Fruit/green vegetable | | | (≥1 time /day) | | | 0.096 |  |  |
| Meat | | | (≥1 time /day) | | | 0.007 | 0.002 | 0.019 |
| Preserved food | | | (≥1 time /day) | | |  | 0.011 |  |
| Alcohol drinking | | Non-drinkers | | | |  |  |  |
|  | | <4 times /week | | | |  |  |  |
|  | | ≥4 times /week | | | | 0.019 | 0.012 | 0.080 |

* Results from univariate logistic regression. *p*<0.10 was showed in table;

^a^ no. of cases / no. of controls;

ETS, environmental tobacco smoke; NA, not applicable.

Appendix table 4 Model performance with or without residential radon exposure (RRE)

| Smoking category | AUC (95% CI)^*^ | | *p* value ^†^ |
| --- | --- | --- | --- |
|  | Without RRE | With RRE |  |
| Never smokers | 0.542 (0.517-0.567) | 0.583 (0.550-0.617) | 0.028 |
| Former smokers | 0.681 (0.646-0.715) | 0.676 (0.641-0.711) | 0.007 |
| Current smokers | 0.532 (0.512-0.553) | 0.535 (0.513-0.557) | 0.543 |

^*^ AUC, area under the curve; CI, confidence interval;

^†^ By DeLong’s test for two ROC curves.

Appendix table 5 Risk predictors and odds ratio for adenocarcinoma of lung by smoking status

|  | | | | | | Odds ratio (95% CI) | | |
| --- | --- | --- | --- | --- | --- | --- | --- | --- |
|  |  |  |  |  |  | Never smokers  (89/536) ^a^ | Former smokers  (124/357) ^a^ | Current smokers  (227/176) ^a^ |
| Age groups  (years) | | <50 | | | |  |  |  |
|  |  | 50-60 | | | |  | 0.20 (0.04-1.04) |  |
|  |  | 60-70 | | | |  | 0.26 (0.06-1.15) |  |
|  |  | ≥70 | | | | 0.57 (0.32-0.99) | 0.14 (0.03-0.63) |  |
| Education | Primary school or below | | | | |  |  |  |
|  | Middle school | | | | |  | 0.47 (0.26-0.85) |  |
|  | College or above | | | | |  | 0.36 (0.19-0.69) | 0.64 (0.41-0.99) |
| Marital status | | | | | Married |  |  |  |
|  |  |  |  |  | Others |  | 0.47 (0.23-0.89) |  |
| History of lung diseases | | | | | | 2.99 (1.48-5.85) | 3.13 (1.84-5.35) |  |
| Cancer history in first-degree relatives | | | | | | 1.82 (1.00-3.25) | 1.59 (0.86-2.89) | 2.67 (1.46-5.09) |
| Smoking and ETS | | | | | | | | |
| Pack-years | | <20 | | | | N.A. |  |  |
|  |  | 20-40 | | | | N.A. | 1.63 (0.91-2.94) | 2.16 (1.08-4.40) |
|  |  | ≥ 40 | | | | N.A. | 2.32 (1.30-4.15) | 2.18 (1.13-4.28) |
| Years of smoking cessation | | | | 2-5 years | | N.A. |  | N.A. |
|  |  |  |  | 5-10 years | | N.A. | 0.45 (0.21-0.89) | N.A. |
|  |  |  |  | 10-20 years | | N.A. | 0.64 (0.36-1.10) | N.A. |
|  |  |  |  | ≥ 20 years | | N.A. |  | N.A. |
| ETS exposure | | | | | | 1.58 (0.92-2.80) |  |  |
| Exposure of indoor air pollutants | | | | | | | | |
| Quantiles of radon | | Q1 (5.17-8.11) | | | |  |  |  |
|  |  | Q2 (8.11-8.64) | | | | 2.26 (1.05-5.06) |  |  |
|  |  | Q3 (8.64-9.77) | | | | 2.05 (0.96-4.57) |  |  |
|  |  | Q4 (9.77-13.00) | | | | 2.78 (1.36-6.03) |  |  |
| Occupational exposure | | | | | | | | |
| Carcinogen exposure | | | | | | 1.94 (1.20-3.17) |  | 1.49 (0.97-2.27) |
| Dust control in workplace | | | | | | 0.36 (0.16-0.87) | 0.19 (0.09-0.40) | 0.25 (0.10-0.56) |
| Dietary habits and drinking habits | | | | | | | | |
| Fruit/green vegetable | | | (≥1 time /day) | | | 1.69 (1.03-2.78) |  |  |
| Meat | | | (≥1 time /day) | | | 0.40 (0.20-0.77) | 0.52 (0.28-0.94) | 0.53 (0.25-1.08) |
| Alcohol drinking | | Non-drinkers | | | |  |  |  |
|  | | <4 times /week | | | |  | 1.57 (0.89-2.79) |  |
|  | | ≥4 times /week | | | | 2.03 (1.03-3.84) | 1.83 (1.04-3.26) |  |

CI, confidential interval; ETS, environmental tobacco smoke; NA, not applicable;

^a^ no. of cases / no. of controls.

Appendix table 6 Risk predictors and odds ratio for squamous cell carcinoma of lung by smoking status

|  | | | | Odds ratio (95% CI) | | |
| --- | --- | --- | --- | --- | --- | --- |
|  |  |  |  | Never smokers  (5/536) ^a^ | Former smokers  (85/357) ^a^ | Current smokers  (128/176) ^a^ |
| Education | Primary school or below | | |  |  |  |
|  | Middle school | | |  | 0.43 (0.23-0.79) |  |
|  | College or above | | |  | 0.24 (0.11-0.49) | 0.39 (0.24-0.64) |
| History of lung diseases | | | |  | 3.27 (1.83-5.84) | 2.06 (1.15-3.76) |
| Cancer history in first-degree relatives | | | |  |  | 2.46 (1.24-5.11) |
| Smoking | | | | | | |
| Pack-years | | <20 | | N.A. |  |  |
|  |  | 20-40 | | N.A. | 2.17 (1.05-4.58) | 3.66 (1.48-10.11) |
|  |  | ≥ 40 | | N.A. | 3.45 (1.79-6.88) | 5.33 (2.27-14.15) |
| Occupational exposure | | | | | | |
| Mask used in workplace | | | |  | 0.37 (0.11-0.97) | 2.44 (1.15-5.43) |
| Dust control in workplace | | | |  | 0.17 (0.07-0.40) | 0.42 (0.15-1.03) |
| Dietary habits and drinking habits | | | | | | |
| Meat | | | (≥1 time /day) |  | 0.60 (0.29-1.19) | 0.45 (0.19-1.00) |

CI, confidential interval; NA, not applicable;

^a^ no. of cases / no. of controls.

Appendix table 7 Risk predictors and odds ratio for small cell lung cancer by smoking status

|  | | | | | Odds ratio (95% CI) | | |
| --- | --- | --- | --- | --- | --- | --- | --- |
|  |  |  |  |  | Never smokers  (0/536) ^a^ | Former smokers  (26/357) ^a^ | Current smokers  (92/176) ^a^ |
| Age groups  (years) | | <50 | | |  |  |  |
|  |  | 50-60 | | |  |  |  |
|  |  | 60-70 | | |  |  | 2.47 (1.03-5.97) |
|  |  | ≥70 | | |  |  | 3.36 (1.70-6.93) |
| Education | Primary school or below | | | |  |  |  |
|  | Middle school | | | |  | 0.44 (0.17-1.21) | 0.51 (0.24-1.07) |
|  | College or above | | | |  | 0.31 (0.08-1.04) | 0.19 (0.08-0.43) |
| Smoking | | | | | | | |
| Pack-years | | <20 | | | N.A. |  |  |
|  |  | 20-40 | | | N.A. | 2.90 (0.81-11.98) | 7.33 (1.97-38.21) |
|  |  | ≥ 40 | | | N.A. | 3.85 (1.23-14.78) | 9.03 (2.60-44.75) |
| Years of smoking cessation | | | | 2-5 years | N.A. |  | N.A. |
|  |  |  |  | 5-10 years | N.A. |  | N.A. |
|  |  |  |  | 10-20 years | N.A. |  | N.A. |
|  |  |  |  | ≥ 20 years | N.A. | 0.39 (0.11-1.12) | N.A. |
| Exposure of indoor air pollutants | | | | | | | |
| Incense burning at home | | | | |  | 3.34 (1.19-11.93) |  |
| Occupational exposure | | | | | | | |
| Mask used in workplace | | | | |  |  | 2.84 (1.04-7.86) |
| Dust control in workplace | | | | |  |  | 0.16 (0.05-0.46) |
| Dietary habits and drinking habits | | | | | | | |
| Preserved food | | | (≥1 time /day) | |  | 2.48 (1.03-5.89) | 2.29 (1.18-4.51) |

CI, confidential interval; NA, not applicable;

^a^ no. of cases / no. of controls.
